# Non-Photochemical Quenching Capacity in *Arabidopsis thaliana* Affects Herbivore Behaviour

**DOI:** 10.1371/journal.pone.0053232

**Published:** 2013-01-02

**Authors:** Hanna Johansson Jänkänpää, Martin Frenkel, Ismayil Zulfugarov, Michael Reichelt, Anja Krieger-Liszkay, Yogesh Mishra, Jonathan Gershenzon, Jon Moen, Choon-Hwan Lee, Stefan Jansson

**Affiliations:** 1 Umeå Plant Science Centre, Department of Plant Physiology, Umeå University, Umeå, Sweden; 2 Department of Ecology and Environmental Science, Umeå University, Umeå, Sweden; 3 Department of Molecular Biology, Pusan National University, Busan, Republic of Korea; 4 Institute of Botany, Azerbaijan National Academy of Sciences, Baku, Azerbaijan; 5 Department of Biochemistry, Max Planck Institute for Chemical Ecology, Jena, Germany; 6 CEA, Institut de Biologie et Technologies de Saclay, Service de Bioénergétique Biologie Structurale et Mécanisme, Gif-sur-Yvette, France; RIKEN Plant Science Center, Japan

## Abstract

Under natural conditions, plants have to cope with numerous stresses, including light-stress and herbivory. This raises intriguing questions regarding possible trade-offs between stress defences and growth. As part of a program designed to address these questions we have compared herbivory defences and damage in wild type *Arabidopsis thaliana* and two “photoprotection genotypes”, *npq4* and *oePsbS*, which respectively lack and overexpress PsbS (a protein that plays a key role in qE-type non-photochemical quenching). In dual-choice feeding experiments both a specialist (*Plutella xylostella*) and a generalist (*Spodoptera littoralis*) insect herbivore preferred plants that expressed PsbS most strongly. In contrast, although both herbivores survived equally well on each of the genotypes, for oviposition female *P. xylostella* adults preferred plants that expressed PsbS least strongly. However, there were no significant differences between the genotypes in levels of the 10 most prominent glucosinolates; key substances in the *Arabidopsis* anti-herbivore chemical defence arsenal. After transfer from a growth chamber to the field we detected significant differences in the genotypes’ metabolomic profiles at all tested time points, using GC-MS, but no consistent “metabolic signature” for the lack of PsbS. These findings suggest that the observed differences in herbivore preferences were due to differences in the primary metabolism of the plants rather than their contents of typical “defence compounds”. A potentially significant factor is that superoxide accumulated most rapidly and to the highest levels under high light conditions in *npq4* mutants. This could trigger changes *in planta* that are sensed by herbivores either directly or indirectly, following its dismutation to H_2_O_2_.

## Introduction

Plants are sedentary organisms and cannot escape unfavourable conditions, thus they have to cope with a host of biotic and abiotic stresses, such as drought, cold, light, herbivores and pathogens. Among the most rapidly changing abiotic factors are light levels. For instance, a passing cloud can change light intensities by orders of magnitude within seconds, causing photooxidative stress. Increases in light intensity increase photosynthetic rates, but at a certain threshold intensity the photosynthetic apparatus becomes saturated and further excitation causes photooxidative damage. The reaction centre of photosystem II is particularly susceptible [1]. Photooxidative damage is caused the excessive production of reactive oxygen species (ROS), such as singlet oxygen, superoxide and hydrogen peroxide [2], in cases where, for example, excited chlorophyll molecules cannot transfer their excitation energy to the reaction centre pigments and instead transfer it to oxygen.

Resistance to high light stress has been thoroughly investigated in studies showing that plants have several potent lines of defence against high light stress, including biochemical responses and movements of both leaves and chloroplasts (see [3], for a review). Their biochemical defences include three types of non-photochemical quenching (NPQ): the qE type, state-transition and photoinhibition [4]. The first of these, qE, seems to substantially affect plant performance under natural conditions [5], [6]. Research in the last decade has elucidated much of the mechanism and players involved in qE [7]. A crucial mediator is the PsbS protein, which catalyses shifts of the light harvesting antenna from an optimal light harvesting state to another allowing harmless dissipation of some excitation energy as heat [8]. Studies on *Arabidopsis* mutants and transgenics with different PsbS levels have also highlighted the importance of photoprotection for plant performance. Mutant plants that lack PsbS (*npq4*) – and thus almost all qE-type NPQ – have shown reduced fitness under natural conditions in the field [5], while plants overexpressing PsbS (oePsbS) have shown a two-fold increase in qE [9]. In recent analyses of field-grown *npq4*, wild type and oePsbS plants we have also observed transcriptomic and metabolomic shifts associated with changes in PsbS levels [10].

Most, if not all, plants produce chemicals that are toxic, decrease palatability or otherwise deter herbivores, thereby reducing the damage they cause. However, this is costly because the production of chemical defences requires the use of resources that could otherwise be used for growth and reproduction. For example, it has been shown that *Arabidopsis* plants with increased levels of chemical defence substances have lower fitness than controls [11]. Many of these chemicals are not present in the plants at all times, but are induced only when they are attacked, thereby reducing the cost of defence (e.g. [12]). Defence can also be induced in parts of the plant that have not been attacked as a result of a systemic acquired response (SAR); this inevitably consumes resources [13]. Studies on wild radish and tobacco have indicated that induced plants produce more seeds and have higher fitness than control plants when attacked, but not when they are not attacked [14], [15], so resistance appears to be beneficial only in the presence of enemies. The main chemical line of herbivore defence in members of the *Brassica* family, including *Arabidopsis*, is believed to be the accumulation of glucosinolates [16], which is stimulated in *Arabidopsis* by jasmonic acid (JA) [17]. Glucosinolates provide defence against herbivores synergistically with myrosinase, through the production of toxic breakdown products, also known as the “mustard oil bomb”. Glucosinolates and myrosinase are stored in separate cellular compartments and come into contact when cells are damaged, for instance through grazing or wounding [18]. Myrosinase then catalyses glucosinolate hydrolysis, resulting in break-down products that include isothiocyanates, which are toxic to non-adapted herbivores [19]. Several specialist herbivores (e.g. *Plutella xylostella*) possess an enzyme called glucosinolate sulfatase that prevents the formation of toxic compounds and so disarms this defence mechanism [18]. However, herbivores respond to the wide spectrum of chemicals in plants, rather than solely attractants and defence compounds thus metabolic changes that are not directly involved in defence may also influence their responses indirectly.

Our previous findings indicate that *npq4* plants lacking PsbS under natural conditions may be in a ‘permanently’ induced defence or “pre-sensitized” state that allows them to respond more rapidly to stress [10], which may explain their relatively low seed set in the field [5]. Hypothetically, this may be due to the accumulation of one or several ROS species, for example singlet oxygen, superoxide or hydrogen peroxide, which may act as (a) signalling compound(s) and interact with components of other “stress pathways”, such as the octadecanoid pathway leading to JA/jasmonate and its derivative methyl jasmonate (MeJa). Accordingly, elevated levels of enzymes of this pathway have been observed in *npq4* plants, at both transcript and protein levels [10], and the JA pathway modulates developmental changes in addition to stress responses [20], [21], [22]. If *Arabidopsis* plants lacking PsbS produce more ROS than wild type counterparts this may be the initial event in a retrograde signal that induces changes in gene expression affecting multiple aspects of plant development and metabolism. As a further indication of the complexity of interactions between growth, development and stress signalling, phytochromes have also been shown to be involved in both biotic and abiotic stress responses [23].

In the study reported here we compared the responses of herbivores to a set of *Arabidopsis* genotypes with the same genetic background, Columbia-0, that produce different levels of PsbS. Co-evolution between hosts and specialist herbivore insects has frequently resulted in herbivore adaptations to the chemical defence compounds of a species or group of species [24]. Indeed, specialist herbivores may even be attracted to volatile defence compounds of their preferred host plants [25]. In contrast, generalist herbivore insects have to cope with multiple defence compounds from several plant families. Thus, responses of different types of herbivores to plant chemical defences differ. For instance, Agrawal [26] found that herbivory responses of wild radish induced by spraying with JA reduced the growth of generalist larvae, but had no effects on the performance of a specialist herbivore on either induced or control plants. Therefore, hypothesizing that generalist and specialist insect herbivores may respond differently to high light-stressed plants with varying capacities for photoprotection, we compared responses to our *Arabidopsis* genotypes of a polyphagous generalist herbivore (*Spodoptera littoralis*) and a specialist (*Plutella xylostella*) that feeds solely on members of the *Brassica* family. In addition, to explore metabolic differences that may be linked to variations in both PsbS levels and herbivore responses we analysed the genotypes’ metabolic profiles and, more specifically, their glucosinolate, superoxide and peroxide levels and production kinetics. In addition, to explore metabolic differences that may be linked to variations in both PsbS levels and herbivore responses we analysed the genotypes’ global metabolic profiles, as well as their levels and production kinetics of glucosinolates, superoxide and peroxide.

## Results

### Feeding and Oviposition Experiments Indicated that Specialist and Generalist Herbivores could Distinguish between Plants Varying in PsbS Levels

**Figure 1:**
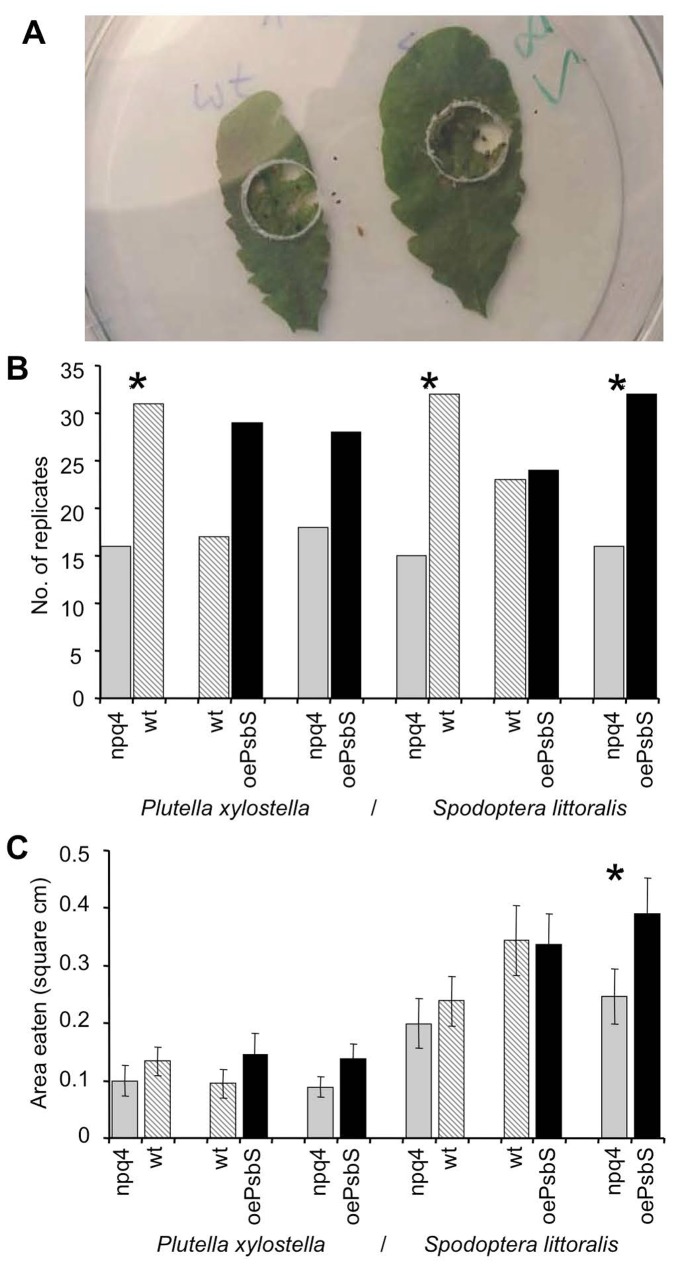
Feeding preferences of *P. xylostella* and *S. littoralis* in dual-choice (cafeteria) tests for three *Arabidopsis* genotypes with differing PsbS levels: *npq4*, wild type (Col) and *oePsbS*. A) Experimental setup showing larval access to plant material. B) Numbers of replicates in which larvae consumed the highest leaf areas of the indicated genotypes; significant differences (binomial tests, p<0.05) indicated by stars. C) Leaf areas consumed (cm^2^), averaged over all days and replicates; significant differences (paired t-tests; p = 0.003) indicated by stars.

First, we compared the food choices of larvae of a specialist (*P. xylostella*) and a generalist (*S. littoralis*) moth in “dual choice” feeding (cafeteria) experiments (Figure 1A). These moths have been used in previous *Arabidopsis* feeding experiments and can be easily reared in the lab. Both herbivore species tended to prefer plants with more PsbS in our dual choice feeding experiments (see Figure 1B, showing that numbers of ‘wins’, i.e. frequencies at which larger areas of their leaves were eaten, were consistently higher for the genotypes with the highest PsbS levels; columns to the right). Binomial tests of the number of ‘wins’ in the pair-wise comparisons confirmed that both herbivores significantly (p<0.05) preferred wild type to *npq4* plants, and the generalist significantly preferred *oePsbS* to *npq4* plants (Figure 1B).

The same pattern was observed when the leaf area consumed in each experiment was analysed rather than merely scoring the ‘winner’ (Figure 1C). In all but one comparison, larger leaf areas were consumed of plants with higher PsbS levels, although the difference was only significant in the *npq4* versus *oePsbS* trial with *S. littoralis* larvae according to a paired t-tests (p = 0.003). Taken together, these results indicate that PsbS levels in the plants influenced the feeding behaviour of both the generalist and specialist herbivore.

Secondly, we studied the performance of larvae after feeding on the three *Arabidopsis* genotypes in the field, to assess whether PsbS levels may indirectly but significantly influence food quality. Equal numbers of *P. xylostella* larvae were hatched on plants of each of the three genotypes and weighed 17 days after the eggs were laid. The larvae on the different genotypes showed no significant differences in weight (ANOVA, F = 0.233, p = 0.793; Figure S1). Similarly, no significant differences were found in the weight of *S. littoralis* larvae fed on different plant genotypes (in cages to prevent predation) in three repeated field experiments during one summer (Figure S2). These findings indicate that both herbivores could successfully utilize *Arabidopsis* leaves with widely differing PsbS levels as food sources.

**Figure 2:**
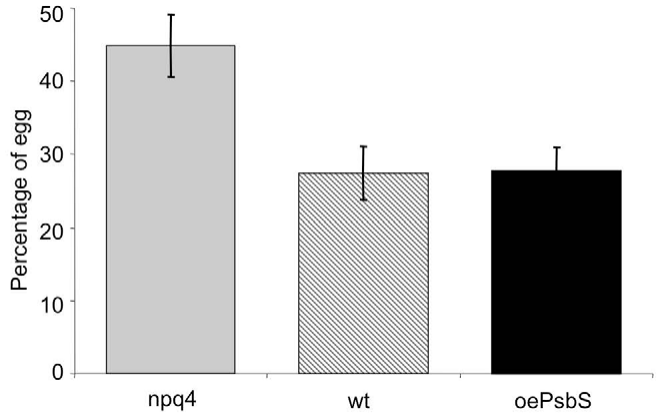
*P. xylostella* oviposition on *Arabidopsis* plants with differing PsbS levels: *npq4*, wild type (Col) and *oePsbS*. All eggs laid over the 10 experimental days were counted, the bars show average percentages laid on each genotype, with standard errors (n = 10).

Thirdly, we studied oviposition by *P. xylostella*. When adult females were allowed to choose between all three genotypes they showed a significant preference (ANOVA, F = 17.758, p = 0.008) to oviposit on the least photoprotected genotype, *npq4* (Figure 2). In all 10 replicates, the number of eggs was highest on *npq4* plants, although the total number of eggs varied between replicates.

### No Differences in Glucosinolate Composition or Concentration/abundance were Detected

These data raise intriguing questions about the biochemical basis of the herbivores’ feeding and oviposition preferences. Reductions in PsbS levels could result in the induction of chemical “defence compounds” that attract specialist *P. xylostella* females for oviposition, but reduce the feeding preferences of both *P. xylostella* and *S. littoralis* larvae. Alternatively, the differences in responses could be linked to changes in primary metabolism, such as shifts in levels of carbohydrates and amino acids, which are also sensitive to growing conditions. Previous studies on these plants were not conclusive in this respect since we have detected both significant differences in primary metabolite profiles of the three genotypes and induction of the JA pathway by low PsbS levels, providing indirect evidence for the induction of defence compounds [10].

**Figure 3:**
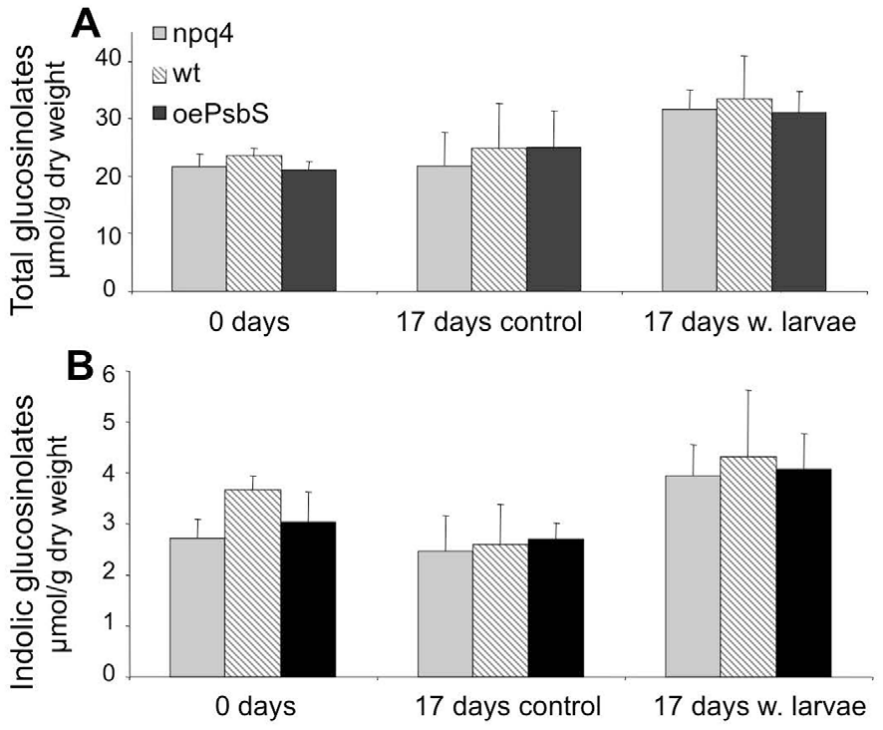
Levels of glucosinolates in leaves of *Arabidopsis* plants with varying PsbS levels. *npq4*, wild type (Col) and *oePsbS*) from the field site, with and without *S. littoralis* larvae at the beginning (after three days of acclimation) and end (17 days later) of the experiment. Error bars are standard deviation, n = 10. A) Total levels of glucosinolates and (B) levels of indolic glucosinolates in the three genotypes after indicated time points/treatments.

We sampled plants both with and without herbivore damage caused by *S. littoralis* from the field, three days (when the larvae experiment started) and 17 days after transfer to the field, then quantified the 10 most prominent glucosinolate (GS) compounds in the samples: 3-Methylsulfinylpropyl glucosinolate (3MSOP), 4-Methylsulfinylbutyl glucosinolate (4MSOB), 5- Methylsulfinylpentyl glucosinolate (5MSOP), 4-Hydroxy-indol-3-yl-methyl glucosinolate (4OHI3M), 7-Methylsulfinylheptyl glucosinolate (7MSOH), 4-Methylthiobutyl glucosinolate (4MTB), Indole-3-yl-methyl glucosinolate (I3M), 8-Methylsulfinyloctyl glucosinolate (8MSOO), 4-Methoxy-indol-3-yl-methyl glucosinolate (4MOI3M) and 1-Methoxy-indol-3-yl-methyl glucosinolate (1MOI3M). No significant difference in either the relative or absolute abundance of any GS between the three genotypes was detected (Figure 3A and Table S1). Further, levels of the three indolic glucosinolates (4OHI3M, I3M, 4MOI3M and 1MOI3M), which are known to be inducible by herbivore damage, increased equally in response to herbivory in all three genotypes (Figure 3B). Therefore, we conclude that the observed differences in herbivore preferences were not due to differences in levels of glucosinolates between the genotypes. As shown in Table S1, the levels of two GS (8MSOO and 4MOI3M) also increased in the control plants.

### PsbS Levels Influenced the Plants’ Amino Acid, Carbohydrate and Organic Acid Profiles

Leaf metabolism is strongly influenced by environmental variables. For example, we have observed highly complex and dynamic metabolomic responses to changes in light levels in parallel gas chromatography–mass spectrometry (GC-MS)-based analyses of *Arabidopsis* plants grown both under different light regimes in climate chambers and outdoors [27]. However, in our previous comparison of the three genotypes (where we detected significant metabolomic differences amongst them) we only sampled at one time point [10]. Therefore, to assess potential environmental effects on the differences, in the present study we sampled whole plant rosettes at numerous time points after cultivation in a climate chamber and subsequent exposure to field conditions, as described in [28]. The experiment was repeated on two occasions, in different years.

**Figure 4:**
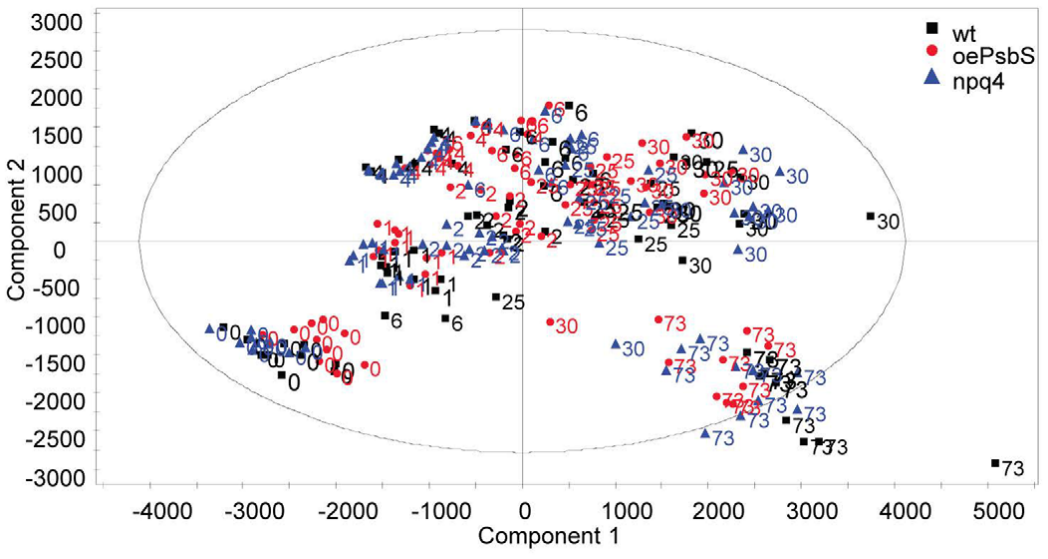
Results of Principal Component Analysis (PCA, score plot) of metabolites in leaves of *Arabidopsis* plants with differing PsbS levels. *Npq4*, wild type (Col) and *oePsbS* after transfer to the field. Genotypes are distinguished by colors and symbols, while the numbers indicate time points when samples were taken. Analyzed with SIMCA-P+12.0.1, R2X [1] = 0.424, R2X [2] = 0.194.

We detected significant differences between the genotypes at almost all individual time points, as in our previous study. However, the between-genotype differences were confounded by huge overall variations in the metabolomic composition of the leaves, associated with factors such as differences in time after transfer and time of the day. When we analysed the whole dataset it was apparent that the sampling time influenced the leaf metabolite profiles much more strongly than genotype (Figure 4). Similarly to our findings in a parallel study of solely wild type plants [27], principal component analysis indicated that changes in growth conditions induced rapid shifts in the leaf metabolite composition along the first principal component, describing 42% of the variation in levels of both identified and non-identified metabolites, while a slower acclimation phase was responsible for 19% of the variation, along the second principal component.

Clearly, metabolic differences between the genotypes occurred at given time points, but these differences were obscured by the huge plasticity in metabolite composition caused by variations in environmental conditions. Therefore, we created a list of identified metabolites significantly separating the wild type and *npq4* plants at any time point. The list contained 35, of 54 identified metabolites in total, and 28 of these 35 had a known molecular identity, most of them being amino acids, carbohydrates or organic acids (see Table 1, which also shows insignificant tendencies, to illustrate the complexity of the responses). However, no metabolite was consistently more or less abundant in *npq4* than in wild type plants. Sucrose levels frequently tended to be lower in *npq4* mutants, but in most cases the differences were not significant, and at one sampling point (30 h) the mean values were almost identical. Fumaric acid levels also tended to be lower in *npq4* mutants at most time points, but the patterns for all other identified metabolites were inconsistent. Likewise, we made a comparison between the levels of these metabolites between wild type and *oePsbS* plants (Table 2). Similarly, sucrose levels tended to be lower in *oePsbS* than in wild type plants, and their homoserine contents were higher at all time points, while the pattern for fumaric acid was inconsistent.

**Table 1:** Metabolites significantly differing between wild type and *npq4* leaves.

Time	0 h	1 h	2 h	4 h	6 h	25 h	30 h	73 h
Metabolites	+ and – indicate that the metabolite was significantly more and less abundant in *npq4* leaves than in wild type leaves, respectively, t-test p<0.05+ and – in brackets () indicate tendencies (p-value, 0.05–0.5)							
Anhydroglucose 1,6	–	0	0	0	(+)	+	+	0
Asparagine	(−)	+	(−)	(+)	–	(−)	0	+
Aspartic acid	0	+	0	(+)	0	(+)	(+)	(−)
B-sitosterol	0	+	(+)	0	(−)	(−)	0	0
Citric acid	(+)	(−)	–	(−)	+	(+)	(+)	0
Fructose	–	(−)	(+)	+	–	–	(−)	(−)
Fumaric acid	(−)	–	(−)	0	0	(−)	0	(−)
Gluconic acid	(−)	(+)	–	(+)	(+)	0	(+)	–
Glucose 6-P	(−)	(+)	(+)	(+)	(−)	–	(−)	(−)
Glutamic acid	–	0	–	(+)	(−)	0	(+)	(−)
Glutamine	(−)	0	(−)	0	(+)	+	(+)	(−)
Glyceric acid	(−)	0	–	–	0	(+)	0	(−)
Glycerol 3-P	0	+	0	0	(+)	0	0	–
Glycine	0	(+)	–	(−)	0	0	0	–
Hexadecanoic acid	0	(+)	0	(−)	+	0	(+)	(−)
Homoserine	(−)	(−)	(−)	–	0	(−)	(+)	(−)
Hydroxybenzoic acid	(−)	(+)	0	(−)	+	(+)	(+)	(−)
Malic acid	(−)	0	–	(−)	–	(−)	(+)	(−)
Myo-inositol	(−)	–	(−)	(+)	+	0	(−)	–
Phenylalanine	–	+	(−)	(+)	+	0	(+)	(−)
Serine	–	0	–	(+)	0	(+)	(+)	(−)
Shikimic acid	(−)	0	–	(−)	(−)	0	(+)	–
Sinapinic acid	(−)	(+)	0	0	(+)	(−)	0	–
Spermidine	0	(+)	0	0	(−)	–	(+)	(−)
Stearic acid	0	(+)	0	(−)	+	0	0	(−)
Sucrose	(−)	(−)	(−)	(−)	–	–	0	(−)
Threonine	–	(+)	–	(+)	(+)	+	+	(−)
Valine	0	(+)	–	0	+	0	(+)	(−)

**Table 2:** Metabolites significantly differing between wild type and oePsbS leaves.

Time	0 h	1 h	2 h	4 h	6 h	25 h	30 h	73 h
Metabolites	+ and – indicate that the metabolite was significantly more and less abundant in oePsbS leaves than in wild type leaves, respectively, t-test p<0.05.+ and – in brackets () indicate tendencies (p-value = 0.05–0.5)							
Anhydroglucose 1,6	+	+	(+)	+	+	–	–	(−)
Asparagine	–	–	–	–	+	+	+	–
Aspartic acid	(−)	–	–	+	+	+	+	–
B-sitosterol	+	+	+	+	–	–	–	+
Citric acid	+	(+)	(+)	+	(−)	+	–	–
Fructose	–	–	+	+	(−)	–	–	–
Fumaric acid	+	(+)	(+)	(+)	–	–	(−)	–
Gluconic acid	+	–	+	–	–	–	–	–
Glucose 6-P	(−)	–	–	–	+	+	+	–
Glutamic acid	(−)	–	–	+	–	+	+	(−)
Glutamine	(−)	(−)	+	–	+	–	–	–
Glyceric acid	–	–	(−)	(−)	+	–	–	–
Glycerol 3-P	–	+	+	–	–	–	–	(−)
Glycine	–	–	–	–	+	+	–	–
Hexadecanoic acid	–	+	+	+	–	(−)	–	–
Homoserine	(−)	–	(−)	(−)	–	–	–	–
Hydroxybenzoic acid	+	–	+	+	–	(−)	–	(−)
Malic acid	(+)	(+)	+	+	–	–	(−)	(−)
Myo-inositol	–	(−)	–	+	(−)	(−)	–	(−)
Phenylalanine	(−)	+	–	–	–	+	+	–
Serine	(−)	(−)	–	+	–	–	–	(−)
Shikimic acid	+	–	–	–	(−)	–	(−)	(−)
Sinapinic acid	–	+	+	+	+	–	(−)	(−)
Spermidine	(−)	–	(−)	–	+	–	+	+
Stearic acid	–	+	+	+	+	(−)	(−)	–
Sucrose	–	(−)	(−)	–	(−)	(−)	(−)	(−)
Threonine	(−)	–	–	–	–	+	–	–
Valine	–	+	–	+	+	–	–	–

Collectively, these data show that differences in PsbS expression had significant effects on leaf metabolism that could be potentially sensed by a grazing herbivore, but the interactions with environmental conditions were very strong, so there was no clear “metabolic signature” of plants either lacking or overexpressing PsbS,

### PsbS Protects from Superoxide Production

**Figure 5:**
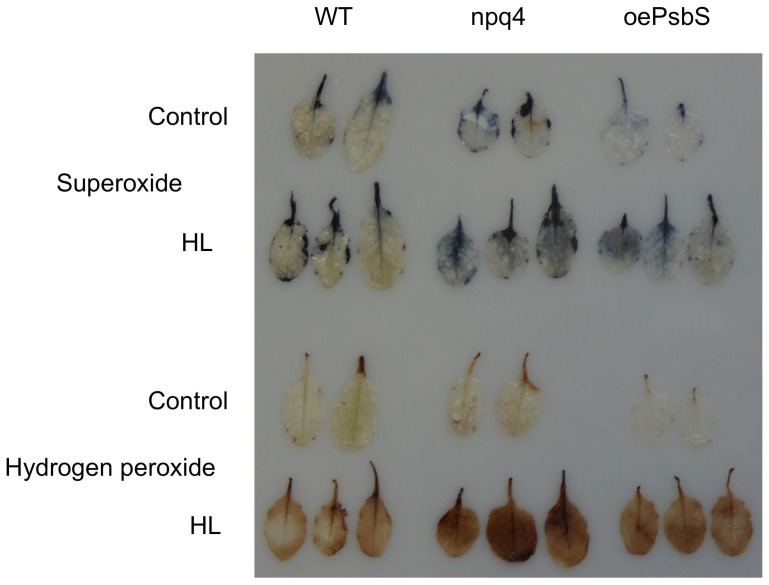
Superoxide and hydrogen peroxide levels in leaves of *Arabidopsis* with differing PsbS levels. Representative images of superoxide and hydrogen peroxide detected histochemically in leaves of *npq4*, wild type (Col) and *oePsbS* plants before and after a 2 hour high light (HL) treatment.

**Figure 6:**
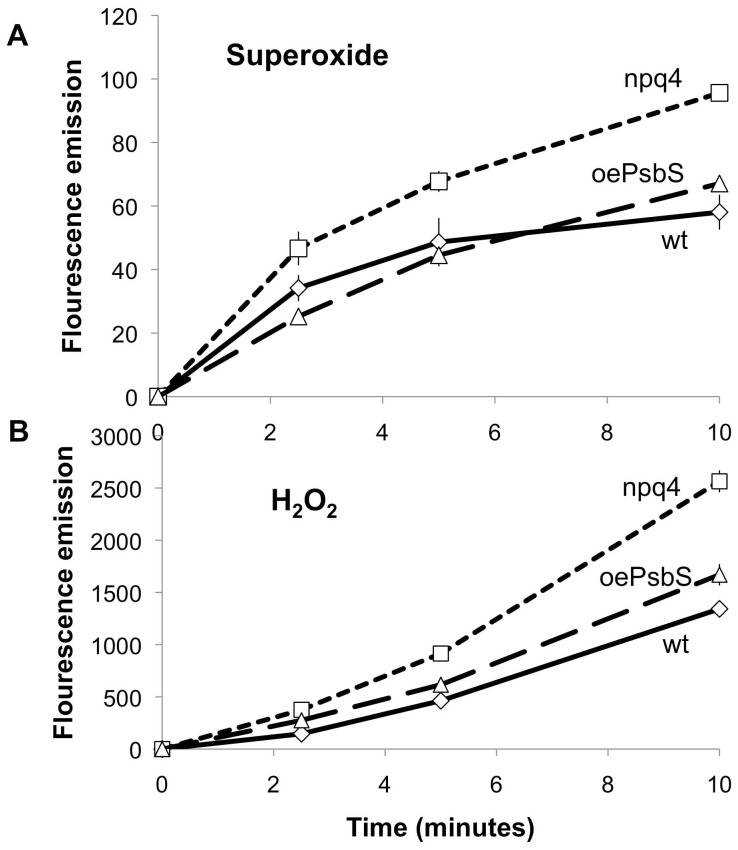
Time courses of superoxide and hydrogen peroxide generation in thylakoids from leaves of *Arabidopsis* plants with differing PsbS levels: *npq4*, wild type (Col) and *oePsbS*. Thylakoid suspensions containing 10 µg chlorophyll per mL prepared from leaves of the plants were illuminated at 700 µmol photons m^−2^s^−1^ at room temperature. A) Fluorescence from dihydroethidium (25 µM) at 590 nm used to detect superoxide production. B) Fluorescence from DCFDA (10 µM) at 525 nm used to detect hydrogen peroxide. Error bars indicate standard deviations, n>3.

Finally, we compared levels and kinetics of superoxide (O_2_^.−^) and hydrogen peroxide (H_2_O_2_) in wild type, *npq4* and *oePsbS* plants, as we have previously shown that rice mutants lacking PsbS produce ROS more abundantly than wild type counterparts (Zulfugarov et al., submitted). We monitored superoxide and hydrogen peroxide production both histochemically and by fluorescence analysis. In dark-adapted samples, the histochemical analysis showed that very little superoxide and hydrogen peroxide was generated in either genotype (Figure 5), but after illumination by high light (1 000 µmol photons m^−2^s^−1^), more superoxide and hydrogen peroxide appeared to be produced in *npq4* than in wild type and *oePsbS* leaves. The fluorescence assay, performed on thylakoids, allowed quantification of the differences and showed that superoxide was produced much more rapidly than hydrogen peroxide (Figure 6); substantial amounts of superoxide accumulated within 2.5 minutes. To confirm this finding with an independent method we used electron paramagnetic resonance (EPR) spin trapping to detect hydroxyl radicals produced from hydrogen peroxide/superoxide in intact leaves. The spin trap signal from *npq4* leaves was 77% stronger than the corresponding signal from wild type leaves (Figure S3). In the presence of an uncoupler that eliminates the proton gradient over the thylakoid membrane, and hence qE, the wild type and *npq4* signals differed by only 4.3%. If leaves were not pre-treated with high light but kept at room light intensity, the signal was only about 7% of the signal obtained after high-light treatment and no difference was detected between the genotypes. Taken together, these data show that *npq4* leaves produced more superoxide and hydrogen peroxide under high light conditions.

## Discussion

Interactions between plants and herbivores are enormously complex. They are strongly influenced by numerous environmental variables and there are strong within- and between-species variations, partly due to intricate co-evolution [24], [25]. Hence, diverse plant factors are likely to affect herbivore preferences, and a key objective of our experiments was to explore causal links (if any) between photosynthetic light harvesting and herbivore responses. For this we used a set of *Arabidopsis* plants differing solely in levels of PsbS protein, which is involved in the regulation of light harvesting, to assess its effects on: interactions between *Arabidopsis* and both a specialist and a generalist herbivore, the plants’ primary and secondary metabolism; and ROS production. We found that both the specialist (*P. xylostella*) and generalist (*S. littoralis*) herbivore preferred to feed on plants with more PsbS. However, the specialist preferred plants with less PsbS for another activity (oviposition), illustrating the complexity of the interactions. We should add that another generalist herbivore that occurred frequently at our experimental site, netted slug (*Deroceras reticulata*), also preferred to feed on plants with more PsbS (data not shown). We initially used *P. xylostella* and *Deroceras* as representatives of specialist and generalist herbivores (see [28]), but since *Deroceras* has not previously been used for such preference tests, in the experiments presented here we used *S. littoralis*.

We have previously demonstrated that PsbS protein levels have major effects on, *inter alia*, plant fitness [5], carbohydrate and anthocyanin levels [29] and both leaf metabolism and transcription [10]. Results of the cited studies suggest that defence responses are stronger under natural conditions in genotypes with less photoprotection (in our case *npq4*<wild type< *oePsbS*) and that JA and/or MeJa levels may be increased in plants lacking PsbS. Therefore, we performed most of the experiments involving insect herbivores under field conditions, despite the difficulties often encountered in such studies (see e.g. [28]).

In the dual-choice feeding experiments both herbivore species preferred the more strongly photoprotected plants, although the leaf area eaten by the specialist did not significantly differ among the genotypes. Chen et al [30] demonstrated that *S. exigua* larvae can detect nutritional differences in various food plants and prefer to feed on plants with higher nutritional quality and similar results have been found for many other herbivores on other plants (e.g. [31]). We have previously shown that carbohydrate metabolism is altered in plants lacking PsbS during later stages of the growth cycle, *inter alia* they produce less starch and sucrose, but more glucose and fructose during seed filling [29]. This may be caused by photo-oxidative damage to the photosynthetic apparatus inhibiting synthesis of primary metabolites (as argued in [29]), and/or redirection of metabolism towards defence (as suggested in [10]). In this contribution, we attempted to distinguish between these possibilities by both comparing levels of defence compounds and time courses of changes in leaf metabolism in the genotypes after transfer to the field. The main finding was that the plants’ metabolic network was more complex than expected. We detected significant differences between the genotypes in (*inter alia*) carbohydrate and amino acid levels, but the patterns were not stable and changed over time. At one sampling time point PsbS-free plants may have had the highest fructose levels, but in the following two they may have had similar and then lower levels.

Since JA is involved in responses to herbivores in many plant families, such as the *Solanaceae* [15], *Asteraceae* [32] and *Brassicaceae* [33], and the strength of induced responses vary with JA concentration [34], we examined levels of both constitutive and induced glucosinolates (GS) in each of our genotypes. However, we found no significant differences in GS levels between them. We cannot exclude the possibility that other defence compounds, such as phenolics [35], were induced to varying degrees in them, but it appears unlikely that the changes in herbivore preferences in plants lacking PsbS were due to changes in secondary metabolism leading to the accumulation of defence compounds. It is also possible that the differences in PsbS levels influenced other variables that may affect herbivore preferences but we did not study. For example, JA has been found to affect phloem parenchyma cell wall invagination in a similar fashion to high light [36]. However, we detected significant differences in soluble metabolites between plants with differing levels of PsbS, in particular metabolites close to primary metabolism, which may explain the difference in herbivore preferences. Contrary to previous indications [10], there was no “metabolic signature” that consistently distinguished *npq4* plants from wild type counterparts, but the decreased capacity for qE type NPQ modified the major fluctuations in primary metabolism induced by changes in environmental conditions sufficiently to group leaf samples from each genotype at most time-points based on their metabolite composition. We suggest that this modification of metabolic composition is sufficient to explain both the specialist and generalist herbivores’ feeding preferences, although we cannot pinpoint any crucial factor for these preferences. Interestingly, many of the metabolites affected by the PsbS level reside within the chloroplast, and could therefore potentially be directly affected by changes in qE capacity.

Similarly, although we could detect a significant difference in oviposition preference for *P.* Similarly, although we detected significant PsbS-related effects on the oviposition preferences of *P. xylostella*, the molecular mechanism is not yet clear. It has been argued that oviposition may be stimulated by the synergistic effects of volatile and/or surface compounds in leaves [37]. However, regardless of the nature of the factors sensed by *P. xylostella* that stimulate oviposition, they appear to be more abundant in plants lacking PsbS. We should add that the oviposition experiment was unsuccessful with *S. littoralis*. This species has an unselective oviposition strategy, *S. littoralis* larvae can survive for a number of days without food (during which they can move to other plants) and in the experiment only a small proportion of *S. littoralis* eggs were laid on experimental plants. Most were laid on the containers used for the experiment.

We believe that the most plausible explanation for our results is that leaf primary metabolism is affected in plants with a low degree of PsbS-dependent photoprotection, *inter alia* their sucrose contents are reduced under some conditions and their amino acids pool is modified. These changes appear to reduce their attractiveness for the generalist herbivore, while respectively increasing and decreasing their attractiveness for the specialist herbivore’s oviposition and feeding.

In addition, we explored ways in which signals may be transferred from PSII, the thylakoid protein complex that is primarily affected by losses of PsbS and qE capacity, to the responses that ultimately affect herbivore preferences. Chloroplasts prepared from *npq4* mutants have been recently shown to produce more singlet oxygen than those prepared from wild type or *oePsbS* [38]. Our data show that *npq4* mutants also produce more superoxide and hydrogen peroxide in high light, and results of our kinetic analysis are consistent with superoxide production being the primary event, as suggested from earlier observations of rice plants lacking PsbS (Zulfugarov et al., submitted). Therefore, the minimal signalling pathway from modified levels of PsbS to changes in herbivore defence presumably involves photosynthetic reaction centres, singlet oxygen, superoxide or hydrogen peroxide, and chloroplast enzymes that participate in amino acid and carbohydrate metabolism. Since changes in the transcription of nuclear genes can also be detected in plants with altered amounts of PsbS under field conditions, the signals are also presumably transmitted to the nucleus and are, therefore, by definition retrograde. From currently available data we cannot discern whether this involves diffusion of hydrogen peroxide from the chloroplast, which it can do [39], with consequent effects on cytoplasmic factors and eventually mRNA levels, or if signals related to changes in metabolism are transmitted from the chloroplast in other ways. *In planta* several retrograde signalling pathways are likely to operate simultaneously (see e.g. [40] for a review), and our previous data provide evidence for the involvement of JA signalling [10]. The molecular details are still obscure, but *npq4* plants seem to be primed to respond more strongly to grazing, and future studies may elucidate the key signals, potentially starting with superoxide production by the photosynthetic electron transport chain.

Photoprotection in plants could potentially be enhanced, for example by increasing expression of PsbS, but beyond a certain level the reduction in photooxidative stress could compromise important inducible defences against herbivores or pathogens and thus cease to be beneficial under some conditions (see e.g. [41]). In addition, a causal link between NPQ capacity and pathogen resistance has been recently suggested [42]. Thus, at the start of this study we hypothesized that there could be intriguing evolutionary trade-offs between photoprotection and biotic stress resistance. Collectively, the results confirm that whole plant level regulation of photosynthetic light harvesting influences trophic interactions between *Arabidopsis* and both specialist and generalist insect herbivores. Evolution may therefore favour plants with different amounts of photoprotection, depending on the nature of the biotic stresses they encounter in their habitats. However, although we have observed significant connections between plants’ capacity for qE type NPQ, metabolism and herbivore preferences, we have not clearly elucidated molecular details of the connections. Since the effects of reducing PsbS levels in *Arabidopsis* and the herbivores tested here were rather weak and context-dependent (for example they were not clearly apparent under controlled growth conditions), we have not found unequivocal support for the hypothesis of an evolutionary trade-off between NPQ and herbivore preferences. However, our data clearly show that the interactions involved are enormously complex, and further experiments, including experiments with plant species that have higher intrinsic levels of NPQ than *Arabidopsis*, will be required to elucidate them.

## Experimental Procedures

### Plant Material

For all experiments, seeds of *Arabidopsis thaliana* genotypes Columbia-0 (wild-type), *npq4-1* [8], and *oePsbS* [9] were sown on nutrient-rich commercial soil (Yrkesplantjord, Weibull). The resulting seedlings were grown in growth chambers under short day conditions (8/16 hours light/dark), until transfer to the field, for the field experiments, and the three lines showed no visible phenotypic differences under the growth conditions employed.

For choice (cafeteria) tests and oviposition experiments seeds were sown in small trays, vernalized for four days at 4°C, and then placed in a growth chamber maintained at around 24°C, and 150 µmol photons m^−2^s^−1^ light intensity. After about two weeks, plants were individually re-potted in 7 cm×7 cm pots. For survival tests and glucosinolate analyses plants were vernalized for three days by chilling (+4°C), and then grown in a growth chamber (light intensity about 200 µmol m^−2^s^−1^, temperature 18–23°C, relative humidity 75%) for 3–4 weeks. A cage formed from tight-mesh netting was placed around each pot before transfer to the field site. The plants were acclimated for three days before the field experiments started. For metabolomic sampling seeds were vernalized by chilling (+4°C) for one day and then grown in a growth chamber (temperature 18–23°C, relative humidity 75%) for 27 days. After about two weeks, these plants were re-potted individually in 7 cm×7 cm pots.

In every field experiment the three genotypes were randomly placed in large trays and placed outside in the botanical garden of the University of Umeå (63°49′N 20°18′E). For a more complete description of the field experiments, see [28].

For ROS determination wild type and *npq4* seedlings were grown in soil in a growth room for one month at an irradiance of 100 µmol m^−2^s^−1^ white light provided by fluorescent lamps in 12 h day/12 h night cycles at 23/18±2°C.

### Insect Rearing

*Spodoptera littoralis* (Boisduval) larvae were grown in a lab maintained at around 23°C and fed on an artificial diet based on wheat germ and casein. Adults mated and oviposited on waxy paper in a container. Egg clusters were cut out and placed on the artificial food. *Plutella xylostella* (L.) adults mated and laid their eggs on oviposition foils (pieces of tin foil that had been dipped in autoclaved cabbage juice) in containers with 10% honey solution. Oviposition foils were then placed on Brassica (Brussels sprout) plants, which the larvae fed on, as feeding on diets other than *Arabidopsis* may be important to prevent acclimation, or even adaptation, to *Arabidopsis* leaves.

### Food Preference (Cafeteria) Experiments

Before the start of the dual-choice (cafeteria) experiments, five to six week-old *Arabidopsis* plants were exposed to natural conditions for at least one week, and started after a few sunny days, thus ensuring that they had experienced light stress. *P. xylostella* larvae were grown on Brussels sprout plants, and 2^nd^ or 3^rd^ instar larvae were used. *S. littoralis* larvae were reared on an artificial diet based on potato. The *S. littoralis* larvae used for the experiments were around **1 cm** **long**.

The experiment was designed to test the herbivores’ choices between fully expanded leaves, of similar-sizes, of pairs of the genotypes, as follows. A moist filter paper was placed on the top of an inverted Petri dish. One leaf of each of the two genotypes to be tested was then placed on the moist filter paper, and covered by the bottom of the Petri dish, in which two 10 mm-diameter holes had been drilled to provide a pre-defined access area (0.78 cm^2^) for the herbivores. One larva was placed in each dish before closing the lid. The experiments were started at around 4 p.m., and pictures were taken after 5, 18 and 24 hours to estimate the eaten areas. Scion Image for Windows (© 2000 Scion Corporation; www.scioncorp.com) was used to estimate the area of each leaf that had been eaten. Pictures from the cafeteria experiments were edited using Adobe Photoshop CS so that the eaten areas were coloured black, and the pictures were then saved in TIFF-format for analysis by the image software. In Scion Image only the greyscale Tiff-pictures were used, a threshold was set so that only the black areas remained visible and the numbers of pixels they covered were estimated as ScionImage-values, which were then converted to cm^2^.

### Larval Growth Experiments

Oviposition foils with *P. xylostella* eggs were placed on *Arabidopsis* plants in the climate chamber for five days. After this period, when the first eggs hatched, the plants were taken to the experimental field site, where six plants of each genotype were placed in a tray covered by mosquito netting. After 11 days in the field the larvae were weighed.

In addition, 60 newly hatched *S. littoralis* larvae were placed on 20 plants of each genotype that had already been exposed to natural light conditions for three days. After 17 days in the field the number of surviving larvae were counted and weighed.

### Oviposition Experiments

Three *Arabidopsis* plants (one of each genotype) were placed in a cage covered by mosquito net, and left for seven days under natural conditions in the field. On each of these days, at around 6 pm (half an hour before the lights were switched of in the 8 h light/16 h dark cycles), the plants from one cage were placed randomly, in oviposition cages containing *P. xylostella* adults and honey solution, in the growth chamber. After 24 hours the plants were replaced with previously untested plants from the field. The number of *P. xylostella* eggs on every plant was counted. Due to differences in the total numbers of eggs per experimental day, the results were calculated as the percentage of eggs laid per genotype.

### Glucosinolate Analysis

After three days of acclimation at the field site 10 plants of each genotype were sampled for glucosinolate (GS) analysis and frozen in liquid nitrogen. Newly hatched larva of *S. littoralis* were placed on plants of each genotype and when they had fed on the plants for 17 days both control and larvae-infested plants were harvested and frozen.

The plant material sampled was freeze-dried for about 48 h and ground in a bead mill, and then 20 mg of each ground sample was weighed into a 96-well plate, extracted and analyzed according to [43]. Briefly, GS were extracted from each sample with 1 ml of 80% methanol solution containing 0.05 mM 4-hydroxybenzyl glucosinolate as an internal standard. After centrifugation, 600 µl portions of the extracts were loaded into wells of a 96-well filter plate filled with DEAE Sephadex A 25. The wells were each washed with 500 µl of 80% methanol, twice with 1 ml of MilliQ water and once with 500 µl of 0.02 M MES buffer (pH 5.2). To cleave the glucosinolates 30 µl of sulfatase solution was added per extract and the plates were incubated at room temperature overnight. Desulfo glucosinolates were eluted with 0.5 ml of MilliQ water into 96 deep well plates and separated using high performance liquid chromatography (Agilent 1100 HPLC system, Agilent Technologies) on a reversed phase C-18 column (LiChrospher RP-18, 250 × 4.6 mm i.d., 5 µm, Merck) with an water-acetonitrile gradient (1.5–5% acetonitrile from 0–6 min, 5–7% acetonitrile from 6–8 min, 7–21% acetonitrile from 8–18 min, 21–29% acetonitrile from 18–23 min, 29–43% acetonitrile from 23–30 min followed by a washing cycle; flow 1.0 ml min-1). Detection was performed with a photodiode array detector and peaks were integrated at 229 nm. To quantify specific glucosinolates we used response factors of 2.0 for aliphatic glucosinolates and 0.5 for indolic glucosinolates [43].

### Metabolomic Profiling

At 10 a.m. of August 3, 2009, complete leaf rosettes of 10 plants of each genotype were sampled and frozen in liquid nitrogen as control samples before (0 hours) plants were transferred to the field. Sampling was then repeated after 1, 2, 4, 6, 25, 30 and 73 hours in the field. Frozen samples were stored at −80°C until they were extracted and analyzed by GC-MS analysis, as described in [27]. The acquired data were processed and analysed using the ChromaTOF, MS Search v.2.0, SIMCA-P+12.0.1 and R2.12.0 software packages.

### Detection of superoxide (O_2_^.−^) and Hydrogen Peroxide (H_2_O_2_) Generation

Histochemical staining to detect superoxide (O_2_^.−^) and hydrogen peroxide (H_2_O_2_) production was conducted as previously described [44]–[46], with some modifications. For superoxide determinations, the leaf samples were immersed in 6 mM NBT solution containing 50 mM sodium phosphate (pH 7.5) for 12 h in the dark. To detect hydrogen peroxide, detached leaves of wild type and mutant plants were immersed in 5 mM DAB solution containing 10 mM MES at pH 3.8 for 12 h in darkness. Both reactions were stopped by soaking the leaves with lacto-glycerol-ethanol (1∶1∶4 v/v/v) and boiling in water for 5 min, then the cleared leaves were preserved in 50% ethanol and photographed.

Superoxide (O_2_^.−^) and hydrogen peroxide (H_2_O_2_) production in isolated thylakoids was determined by fluorescence analysis, as follows. First, leaves of one-month-old seedlings were submerged in fluorescent sensor solution for 12–14 h infiltration in the dark at 22°C. Thylakoids from the leaves were then isolated according to [47] and incubated (at a final chlorophyll concentration of 10 µg per mL) in a reaction buffer containing 0.1 M sucrose, 10 mM NaCl, 10 mM KCl, 5 mM MgCl_2_, 10 mM Tricine, 1 mM KH_2_PO_4_ and 0.2% BSA, pH 8.0. In addition, 30 mM sodium ascorbate and 50 µM methyl viologen were added immediately prior to the experiments to mediate deepoxidation of violaxanthin to zeaxanthin and linear electron transport, respectively. Finally, two ROS sensors were added: 25 µM dihydroethidium (DHE) to detect superoxide [48]–[50] and 10 µM 2′,7′-dichlorofluorescein diacetate (DCFDA) to detect hydrogen peroxide [51]. The samples were illuminated at 700 µmol photons m^−2^s^−1^ to ensure photoinhibition, and fluorescence spectra from the ROS sensors were acquired by an F-4500 fluorescence spectrophotometer (Hitachi, Japan).

### Room-Temperature Spin-Trapping EPR Measurements

Spin-trapping assays with 4-pyridyl-1-oxide-N-tert-butylnitrone (4-POBN) (Sigma-Aldrich) were carried out using leaf disks. Leaf disks were vacuum-infiltrated with 20 mM phosphate buffer, pH 6.5, containing the spin trap reagents and then floated on the same buffer while they were illuminated for 1 h with white light (500 µmol photons m^−2^s^−1^) in the presence of 50 mM 4-POBN, 4% ethanol and 50 µM Fe-EDTA. When required, 20 µM nigericin was added as an uncoupler prior to the illumination. EPR spectra were recorded at room temperature in a standard quartz flat cell using an E-scan spectrometer (Bruker, Rheinstetten, Germany), with the following settings: microwave frequency, 86 GHz; modulation amplitude, 1 G; microwave power, 14 mW; receiver gain, 2*103; time constant, 5.1 ms; number of scans, 4.

## Supporting Information

Figure S1 **Weight of** ***P. xylostella*** **larvae after feeding on** ***Arabidopsis*** **plants with differing PsbS levels.** Larvae were weighed 17 days after the eggs were laid. Weight is given in milligrams, error bars indicate standard deviations: *npq4* n = 47, wild type n = 43, oePsbS n = 37.
(PPT)

Figure S2 **Weight of** ***Spodoptera littoralis*** **larvae after feeding on** ***Arabidopsis*** **plants with differing PsbS levels.** Larvae were weighed after 17 days; weight is given in milligrams.Error bars indicate standard deviations, n>15.
(TIF)

Figure S3 **Light-induced hydroxyl radical formation in wt and** ***npq4*** **leaf disks, detected by indirect spin trapping with 4-POBN**. After infiltration with 1 ml of a 4-POBN/ethanol/FeEDTA solution leaf disks were incubated in the same medium for 1 h in the light (500 µmol photons m^−2^s^−1^) before detecting radicals in the medium. Typical EPR spectra of the 4-POBN/α-hydroxyethyl adduct are shown.
(TIF)

Table S1 **Average levels of all glucosinolates measured in micromol per gram dry weight.** Numbers of replicates were ≥8 and +/− indicates the standard deviation.
(TIF)

## References

[1] Long S, Zhu X-G, Naidu S, Ort D (2006) Can improvement in photosynthesis increase crop yields? Plant, Cell & Environment 29: 315–330. 10.1111/j.1365-3040.2005.01493.x 17080588

[2] Havaux M, Niyogi KK (1999) The violaxanthin cycle protects plants from photooxidative damage by more than one mechanism. Proceedings of the National Academy of Sciences 96: 8762–8767. 10.1073/pnas.96.15.8762 PMC17590 10411949

[3] Horton P, Ruban A, Walters R (1996) Regulation of light harvesting in green plants. Annual Review of Plant Physiology and Plant Molecular Biology 47: 655–684. 10.1146/annurev.arplant.47.1.655 15012304

[4] Müller P, Li X-P, Niyogi KK (2001) Non-Photochemical Quenching. A response to excess light energy. Plant Physiology 125: 1558–1566. 11299337 10.1104/pp.125.4.1558 PMC1539381

[5] Kulheim C, Ågren J, Jansson S (2002) Rapid regulation of light harvesting and plant fitness in the field. Science 297. 10.1126/science.1072359 12098696

[6] Frenkel M, Bellafiore S, Rochaix J-D, Jansson S (2006) Hierarchy amongst photosynthetic acclimation responses for plant fitness. Physiologia Plantarum 129: 455–459.

[7] Niyogi K, Li X-P, Rosenberg V, Jung H-S (2004) Is PsbS the site of non-photochemical quenching in photosynthesis? Journal of Experimental Botany 56: 375–382. 15611143 10.1093/jxb/eri056

[8] Li X-P, Björkman O, Shih C, Grossman A, Rosenquist M, et al (2000) A pigment-binding protein essential for regulation of photosynthetic light harvesting. Nature 403: 391–395. 10667783 10.1038/35000131

[9] Li X-P, Müller-Moulé P, Gilmore A, Niyogi K (2002) PsbS-dependent enhancement of feedback de-excitation protects photosystem II from photoinhibition. Proceedings of the National Academy of Sciences 99: 15222–15227. 10.1073/pnas.232447699 PMC137571 12417767

[10] Frenkel M, Kulheim C, Johansson Jänkänpää H, Skogstrom O, Dall'Osto L, et al (2009) Improper excess light energy dissipation in *Arabidopsis* results in a metabolic reprogramming. BMC Plant Biology 9: 12. 19171025 10.1186/1471-2229-9-12 PMC2656510

[11] Cipollini D (2002) Does competition magnify the fitness costs of induced responses in Arabidopsis thaliana? A manipulative approach. Oecologia 131: 514–520. 28547545 10.1007/s00442-002-0909-5

[12] Siemens D, Garner S, Mitchell-Olds T, Callaway R (2002) Cost of defense in the context of plant competition: *Brassica rapa* may grow and defend. Ecology 83: 505–517.

[13] Heil M (2001) The ecological concept of costs of induced systemic resistance (ISR). European Journal of Plant Pathology 107: 137–146.

[14] Agrawal A (1998) Induced responses to herbivory and increased plant performance. Science 279: 1201–1202. 9469809 10.1126/science.279.5354.1201

[15] Baldwin IT (1998) Jasmonate-induced responses are costly but benefit plants under attack in native populations. Proceedings of the National Academy of Sciences 95: 8113–8118. 10.1073/pnas.95.14.8113 PMC20938 9653149

[16] Fahey J, Zalcmann A, Talalay P (2001) The chemical diversity and distribution of glucosinolates and isothiocyanates among plants. Phytochemistry 56: 5–51. 11198818 10.1016/s0031-9422(00)00316-2

[17] Kliebenstein DJ, Kroymann J, Mitchell-Olds T (2005) The glucosinolate-myrosinase system in an ecological and evolutionary context. Current Opinion in Plant Biology 8: 264–271. 15860423 10.1016/j.pbi.2005.03.002

[18] Ratzka A, Vogel H, Kliebenstein D, Mitchell-Olds T, Kroymann J (2002) Disarming the mustard oil bomb. Proceedings of the National Academy of Sciences 99: 11223–11228. 10.1073/pnas.172112899 PMC123237 12161563

[19] Li Q, Eigenbrode S, Stringam G, MR T (2000) Feeding and growth of *Plutella xylostella* and *Spodoptera eridania* on *Brassica juncea* with varying glucosinolate concentrations and myrosinase activities. Journal of Chemical Ecology 26: 2401–2419.

[20] Wasternack C, Miersch O, Kramell R, Hause B, Ward J, et al (1998) Jasmonic acid: biosynthesis, signal transduction, gene expression. Lipid/Fett 100: 139–146.

[21] Mewis I, Tokuhisa J, Schultz J, Appel H, Ulrichs C, et al (2006) Gene expression and glucosinolate accumulation in Arabidopsis thaliana in response to generalist and specialist herbivores of different feeding guilds and the role of defense signaling pathways. Phytochemistry 67: 2450–2462. 17049571 10.1016/j.phytochem.2006.09.004

[22] Jung C, Lyou SH, Yeu S, Kim MA, Rhee S, et al (2007) Microarray-based screening of jasmonate-responsive genes in Arabidopsis thaliana. Plant Cell Reports 26: 1053–1063. 17297615 10.1007/s00299-007-0311-1

[23] Carvalho RF, Campos ML, Azevedo RA (2011) The role of phytochrome in stress tolerance. Journal of Integrative Plant Biology 53: 920–929. 22040287 10.1111/j.1744-7909.2011.01081.x

[24] Gardner S, Agrawal A (2002) Induced plant defence and the evolution of counter-defences in herbivores. Evolutionary Ecology Research 4: 1131–1151.

[25] Mello M, Silva-Filho M (2002) Plant-insect interactions: an evolutionary arms race between two distinct defense mechanisms. Brazilian Journal of Plant Physiology 14: 71–81.

[26] Agrawal A (1998) Induced responses to herbivory in wild radish: Effects on several herbivores and plant fitness. Ecology 80: 1713–1723.

[27] Jänkänpää HJ, Mishra Y, Schröder WP, Jansson S (2012) Metabolic profiling reveals metabolic shifts in Arabidopsis plants grown under different light conditions. Plant, Cell & Environment doi: 10.1111/j.1365–3040.2012.02519.x. 10.1111/j.1365-3040.2012.02519.x 22497620

[28] Frenkel M, Johansson Jänkänpää H, Moen J, Jansson S (2008) An illustrated gardener's guide to transgenic *Arabidopsis* field experiments. New Phytologist 180: 545–555. 18721164 10.1111/j.1469-8137.2008.02591.x

[29] Kulheim C, Jansson S (2005) What leads to reduced fitness in non-photochemical quenching mutants? Physiologia Plantarum 125: 202–211.

[30] Chen Y, Ruberson J, Olson D (2008) Nitrogen fertilization rate affects feeding, larval performance, and oviposition preference of the beet armyworm, *Spodoptera exigua*, on cotton. Entomologia Experimentalis et Applicata 126: 244–255.

[31] Stockhoff B (1993) Protein intake by gypsy moth larvae on homogeneous and hetergeneous diets. Physiological Entomology 18: 409–419.

[32] Van Kleunen M, Ramponi G, Schmid B (2003) Effects of herbivory simulated by clipping and jasmonic acid on *Solidago canadensis* . Basic and Applied Ecology 5: 173–181.

[33] Lu Y-b, Liu S-s, Liu Y-q, Furlong MJ, Zalucki MP (2004) Contrary effects of jasmonate treatment of two closely related plant species on attraction of and oviposition by a specialist herbivore. Ecology Letters 7: 337–345.

[34] Baldwin IT (1996) Methyl jasmonate-induced nicotine production in *Nicotiana attenuate*: Inducing defence in the field without wounding. Entomologia Experimentalis et Applicata 80: 353–364.

[35] Verhage A, Vlaardingerbroek I, Raaijmakers C, Van Dam N, Dicke M, et al. (2011) Rewiring of the jasmonate signaling pathway in Arabidopsis during insect herbivory. Frontiers in Plant Science 2. 10.3389/fpls.2011.00047 PMC3355780 22645537

[36] Amiard Vr, Demmig-Adams B, Mueh KE, Turgeon R, Combs AF, et al (2007) Role of light and jasmonic acid signaling in regulating foliar phloem cell wall ingrowth development. New Phytologist 173: 722–731. 17286821 10.1111/j.1469-8137.2006.01954.x

[37] Spencer J (1996) Waxes enhance *Plutella xylostella* oviposition in response to sinigrin and cabbage homogenates. Entomologia Experimentalis et Applicata 81: 165–173.

[38] Roach T, Krieger-Liszkay A (2012) The role of the PsbS protein in the protection of photosystems I and II against high light in Arabidopsis thaliana. Biochem Biophys Acta (in press). 10.1016/j.bbabio.2012.09.011 23000078

[39] Mubarakshina MM, Ivanov BN, Naydov IA, Hillier W, Badger MR, Krieger-Liszkay A (2010) Production and diffusion of chloroplastic H_2_O_2_ and its implication to signalling. J Exp Bot. 61: 3577–87. 10.1093/jxb/erq171 20595239

[40] Fernández AP, Strand Å (2008) Retrograde signaling and plant stress: plastid signals initiate cellular stress responses. Current Opinion in Plant Biology 11: 509–513. 18639482 10.1016/j.pbi.2008.06.002

[41] Karpinski S, Gabrys H, Mateo A, Karpinska B, Mullineaux PM (2003) Light perception in plant disease defence signalling. Current Opinion in Plant Biology 6: 390–396. 12873535 10.1016/s1369-5266(03)00061-x

[42] Göhre V, Jones AM, Sklenář J, Robatzek S, Weber AP (2012) Molecular crosstalk between PAMP-triggered immunity and photosynthesis. Molecular Plant-Microbe Interactions. 25: 1083–92. 10.1094/MPMI-11-11-0301 22550958

[43] Burow M, Müller R, Gershenzon J, Wittstock U (2006) Altered glucosinolate hydrolysis in genetically engineered Arabidopsis thaliana and its influence on the larval development of *Spodoptera littoralis* . Journal of Chemical Ecology 32: 2333–2349. 17061170 10.1007/s10886-006-9149-1

[44] Fryer M, Oxborough K, Mullineaux P, Baker N (2002) Imaging of photo-oxidative stress responses in leaves. Journal of Experimental Botany 53: 1249–1254. 11997373

[45] Kariola T, Brader G, Li J, Palva E (2005) Chlorophyllase 1, a damage control enzyme, affects the balance between defense pathways in plants. The Plant Cell 17: 282–294. 15598807 10.1105/tpc.104.025817 PMC544505

[46] Mahalingam R, Jambunathan N, Gunjan S, Faustin E, Weng H, et al (2006) Analysis of oxidative signaling induced by ozone in Arabidopsis thaliana. Plant, Cell & Environment 29: 1357–1371. 10.1111/j.1365-3040.2006.01516.x 17080957

[47] Gilmore AM, Shinkarev VP, Hazlett TL (1998) Govindjee (1998) Quantitative analysis of the effects of intrathylakoid pH and xanthophyll cycle pigments on chlorophyll a fluorescence lifetime distribution and intensity in thylakoids. Biochemistry 37: 13582–13593. 9753445 10.1021/bi981384x

[48] Hideg É, Barta C, Kálai T, Vass I, Hideg K, et al (2002) Detection of singlet oxygen and superoxide with fluorescent sensors in leaves under stress by photoinhibition or UV radiation. Plant and Cell Physiology 43: 1154–1164. 12407195 10.1093/pcp/pcf145

[49] Kálai T, Hankovszky OH, Hideg É, Jekö J, Hideg K (2002) Synthesis and structure optimalization of double (fluorescent and spin) sensor molecules. ARKIVOC: 112–120.

[50] Georgiou A, Papapostolou I, Patsoukis N, Tsegenidis T, Sideris T (2005) An ultrasensitive fluorescent assay for the in vivo quantification of superoxide radical in organisms. Analytical Biochemistry 347: 144–151. 16246291 10.1016/j.ab.2005.09.013

[51] Hempel S, Buettner G, O'Malley Y, Wessel D, Flaherty D (1999) Dihydrofluorescein diacetate is superior for detecting intracellular oxidants: Comparison with 2′7′-dichlorodihydrofluorescein diacetate, 5 (and 6)-carboxy-2′7′-dichlorodihydrofluorescein diacetate, and dihydrorhodamine 123. Free Radical Biology and Medicine 270: 146–159. 10.1016/s0891-5849(99)00061-1 10443931

