# Supplementary figures and images for: Non-Photochemical Quenching Capacity in Arabidopsis thaliana Affects Herbivore Behaviour
Source: PLoS One. 2013 Jan 2;8(1):e53232. doi: 10.1371/journal.pone.0053232 (PMC3534670; doi:10.1371/journal.pone.0053232)

## Slide 1
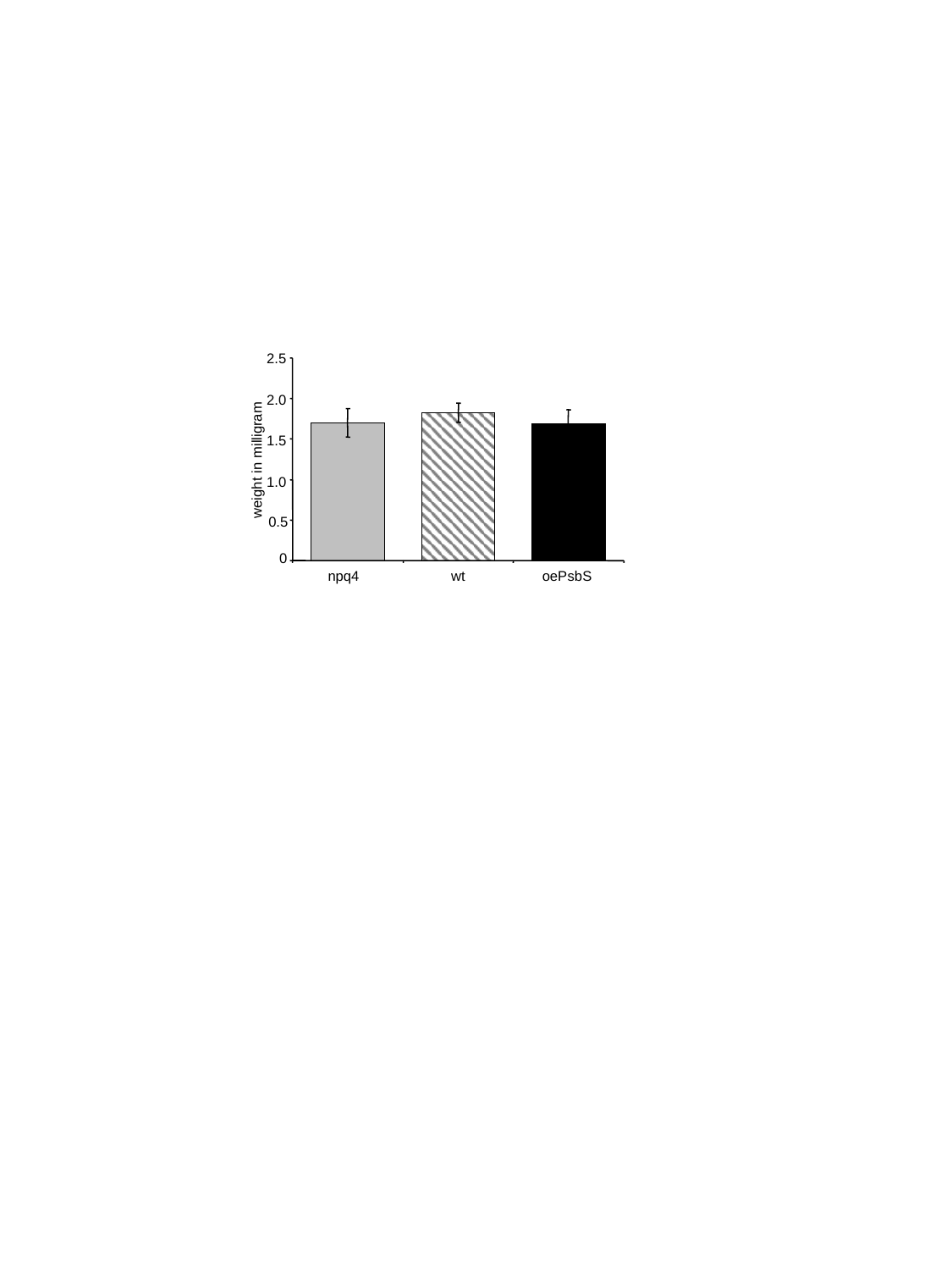

2.5
2.0
1.5
weight in milligram
1.0
0.5
0
npq4
wt
oePsbS

Supplement: Figure S1 — Weight of P. xylostella larvae after feeding on Arabidopsis plants with differing PsbS levels. Larvae were weighed 17 days after the eggs were laid. Weight is given in milligrams, error bars indicate standard deviations: npq4 n = 47, wild type n = 43, oePsbS n = 37. (PPT) [file pone.0053232.s001.ppt]

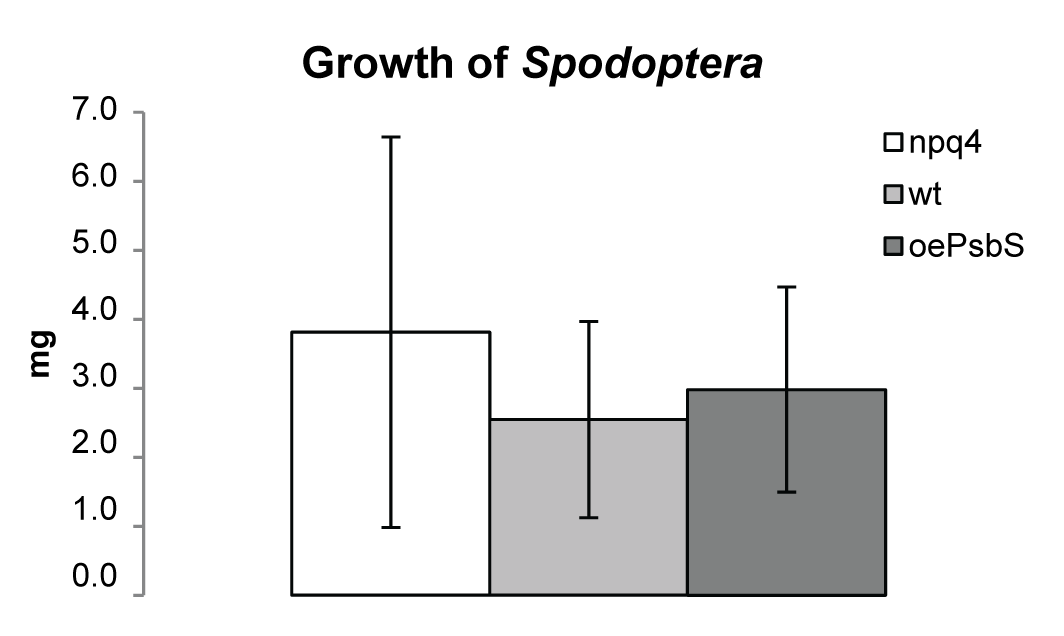

Supplement: Figure S2 — Weight of Spodoptera littoralis larvae after feeding on Arabidopsis plants with differing PsbS levels. Larvae were weighed after 17 days; weight is given in milligrams.Error bars indicate standard deviations, n>15. (TIF) [file pone.0053232.s002.tif]

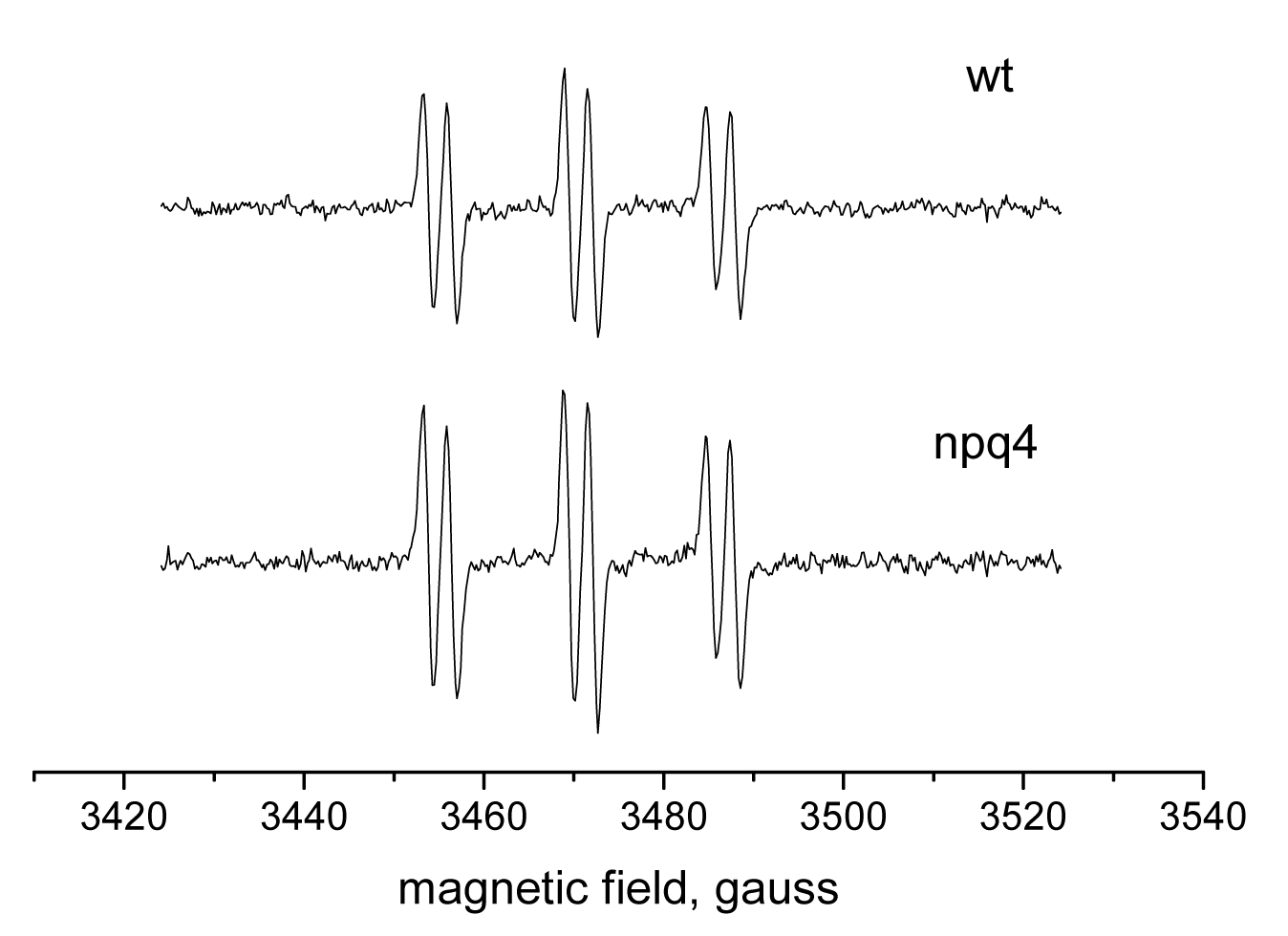

Supplement: Figure S3 — Light-induced hydroxyl radical formation in wt and npq4 leaf disks, detected by indirect spin trapping with 4-POBN. After infiltration with 1 ml of a 4-POBN/ethanol/FeEDTA solution leaf disks were incubated in the same medium for 1 h in the light (500 µmol photons m−2s−1) before detecting radicals in the medium. Typical EPR spectra of the 4-POBN/α-hydroxyethyl adduct are shown. (TIF) [file pone.0053232.s003.tif]

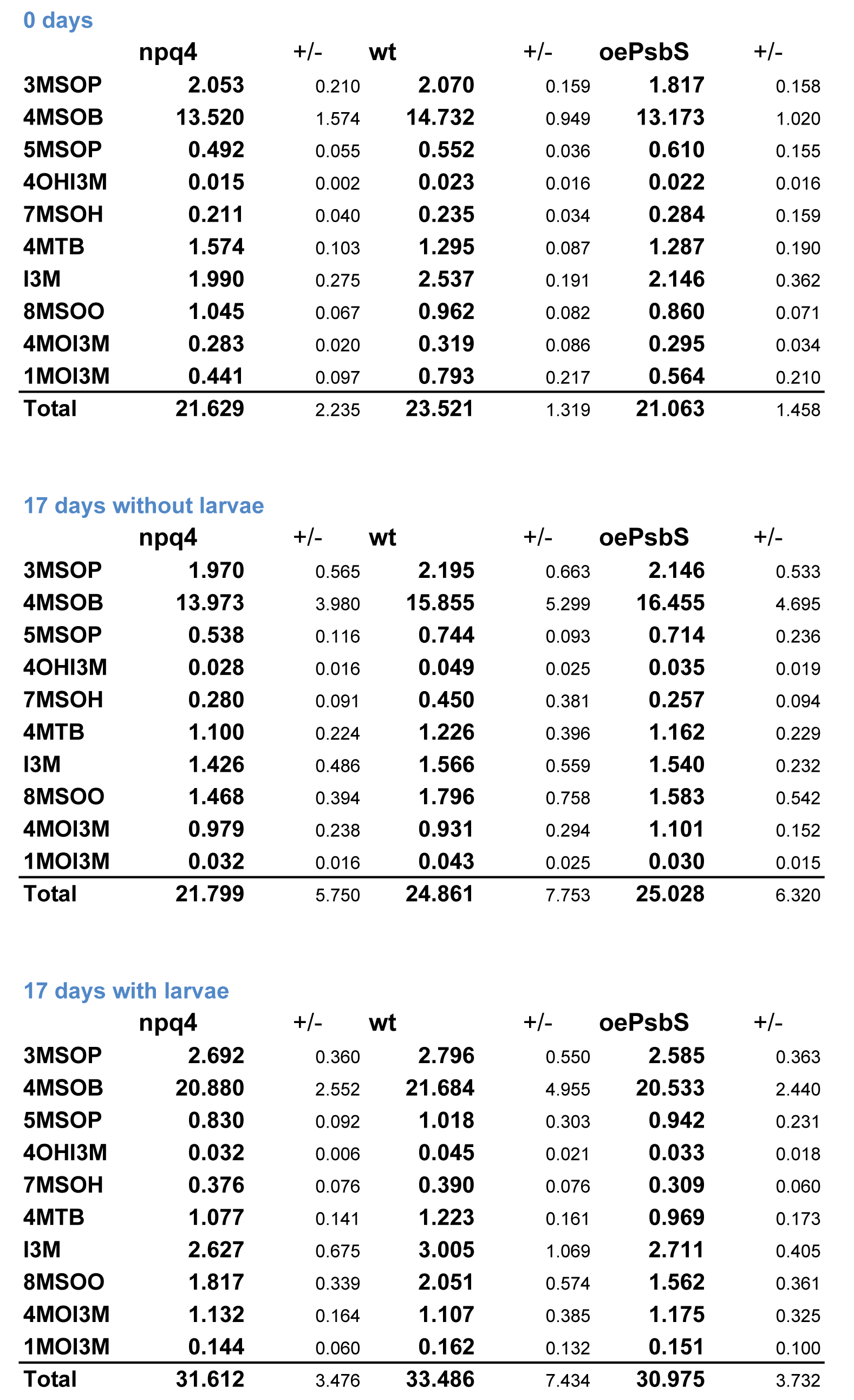

Supplement: Table S1 — Average levels of all glucosinolates measured in micromol per gram dry weight. Numbers of replicates were ≥8 and +/− indicates the standard deviation. (TIF) [file pone.0053232.s004.tif]
